# The effect of education with WhatsApp application on women's health beliefs, self-efficacy levels, and regular mammography behavior: a randomized controlled experimental trial

**DOI:** 10.1590/1806-9282.20250861

**Published:** 2025-10-27

**Authors:** Hasret Yalçınöz Baysal, Sonay Bilgin, Abdullah Baysal, Selen Özdemir, Kemal Yaran, Gülsüm Aşan

**Affiliations:** 1 Ataturk University, Faculty of Nursing, Department of Public Health Nursing – Erzurum, Turkiye.; 2 Erzurum Provincial Health Directorate – Erzurum, Turkiye.; 3 Ataturk University, Institute of Health Sciences – Erzurum, Turkiye.

**Keywords:** Health belief model, Nursing, Mammography, Telemedicine, Self efficacy

## Abstract

**OBJECTIVE:**

The aim of this study was to determine the effect of the Health Belief Model-based training provided to women via WhatsApp on their health beliefs about mammography, self-efficacy levels, and regular mammography behavior.

**METHODS:**

This randomized controlled experimental trial was conducted with 81 women (41 experimental and 40 control), aged 40–69 years, who were literate, able to use WhatsApp, and had no communication barriers, and had no history of breast cancer. Participants were recruited from a Cancer Early Diagnosis, Screening, and Education Center in eastern Turkey. The experimental group was sent training content via WhatsApp once a week for 7 weeks, individual counseling was provided, and the training video was shared in the last week. Data were collected using the "Questionnaire on Descriptive Characteristics," "Health Belief Model Scale for Breast Cancer Screening," and "Mammography Self-Efficacy Scale."

**RESULTS:**

The demographic characteristics of the two groups were similar (p>0.05). After the training, it was determined that women in the experimental group had significantly higher averages in all sub-dimensions of the Health Belief Model Scale for Breast Cancer Screening and Mammography Self-Efficacy Scale compared to the control group (p<0.05). It was also observed that 70% of the women in the experimental group underwent mammography after the training.

**CONCLUSION:**

Health Belief Model-based education delivered via WhatsApp positively affected women's health beliefs and mammography behaviors, demonstrating the effectiveness of education delivered through digital platforms.

## INTRODUCTION

Breast cancer is the most common cancer among women, and early diagnosis significantly improves life expectancy^1^. Mammography can reduce mortality by up to 30%^2^, yet regular screening rates in women over the age of 40 years remain low^3^. In Turkey, this rate ranges from 15 to 32%, compared to 52.7–84% in European countries^4,5^. National guidelines recommend biennial mammography for women aged 40–69 years, annual clinical breast exams, and monthly breast self-examinations^6^. However, awareness and participation in these practices remain inadequate^7–9^.

Among behavioral models, the Health Belief Model (HBM) plays a central role in promoting breast cancer awareness and screening^7–9^. The HBM explains health behavior through five key constructs: susceptibility to disease (sensitivity), perceived severity of its outcomes (seriousness), motivation to protect one's health (health motivation), perceived benefits of screening, and perceived barriers that hinder such behavior^7,8,10^. Studies show that HBM-based interventions significantly increase sensitivity, seriousness, health motivation, and perceived benefits while reducing perceived barriers^11–13^. Another important factor is self-efficacy, referring to a person's belief in their ability to carry out a behavior. Individuals with high self-efficacy are more persistent in adopting and maintaining health behaviors^14^. Research further shows that conventional education can enhance mammography self-efficacy among women^15–17^.

Mobile technologies have emerged as effective health education tools due to their accessibility and ability to overcome logistical barriers^18^. WhatsApp, a widely used platform, supports multimedia communication and group interaction, increasing educational engagement^19^. Studies suggest that WhatsApp-based education improves health beliefs, self-efficacy, knowledge, and behaviors such as self-breast examination^19,20^. However, its effect on mammography behavior remains underexplored^19,20^. This study therefore aimed to assess the impact of HBM-based education via WhatsApp on women's mammography-related health beliefs, self-efficacy, and screening behavior.

### Research hypotheses

H_1_: Education and counseling provided through the WhatsApp application about breast cancer and mammography affect women's health beliefs.

H_2_: Education and counseling provided through the WhatsApp application about breast cancer and mammography will increase women's mammography self-efficacy level.

H_3_: Education and counseling provided through the WhatsApp application about breast cancer and mammography affect women's adherence to regular mammography screening.

## METHODS

### Study design and participants

The study was designed as a randomized controlled experimental trial. The population included women aged 40–69 years who applied to the Cancer Early Diagnosis, Screening, and Education Center in eastern Turkey, had undergone mammography, had accessible phone contact information, and had scheduled appointments as of December 2022. Based on the findings of Chalambari et al.^9^, who reported a high effect size (d=0.90) in similar HBM-based interventions, the required sample size was calculated using G*Power 3.1 with 95%CI, 5% margin of error, and an effect size of 0.90, resulting in a minimum of 68 participants (34 per group). Of the 500 women reached by phone, 81 agreed to participate and were randomly assigned to the experimental (n=41) and control (n=40) groups using the "Research Randomizer." The study collected data from 41 experimental and 40 control group participants.

### Inclusion and exclusion criteria

Women between the ages of 40 and 69 years who had no diagnosis or history of breast cancer, were literate and communicative, able to use WhatsApp, had not undergone a mammogram in the past 2 years, and volunteers were included in the study; women who had previously experienced problems with breast cancer, had regular mammograms, or had undergone breast-conserving surgery were excluded.

### Data collection forms

- Questionnaire on descriptive characteristics: The questionnaire on descriptive characteristics, developed by the researchers in line with the literature, consisted of 15 questions covering sociodemographic characteristics and the health history of women^7,12^.
- Health Belief Model Scale for Breast Cancer Screening: The scale, developed by Champion and revised in 1999, measures beliefs and attitudes regarding breast cancer, breast self-examination, clinical breast examination, and mammography^21^. It was adapted into Turkish by Gözüm and Aydın^10^ and includes eight subscales with 52 items. This study used five subscales: "sensitivity, seriousness, health motivation, mammography benefits, and mammography barriers." The 5-point Likert scale is scored separately for each subscale, with higher scores indicating stronger beliefs. In the original study, Cronbach's alpha reliability coefficients for the scale ranged from 0.69 to 0.81^21^. In this study, the Cronbach alpha coefficients for the subscales ranged between 0.82 and 0.88 in the pre-test and between 0.81 and 0.91 in the post-test.
- Mammography Self-Efficacy Scale: The scale developed by Champion, Skinner, and Menon^22^ was developed by Seçginli^14^. The Turkish version of the scale was validated, and its reliability was confirmed. The scale assesses perceived self-efficacy about the process of mammography. It is a Likert-type scale with 10 items scored from 1 to 5. A higher score for each dimension indicates a higher likelihood of having a mammogram. The Cronbach's alpha value of the Turkish version of the scale was found to be 0.90^14^. In our study, the Cronbach's alpha value of the scale was found to be 0.83 for the pre-test and 0.92 for the post-test.

### Data collection

Data were collected between December 2023 and June 2024 by contacting women who had not yet completed their scheduled mammography appointments and obtaining their voluntary consent. The data collection tools were administered online via Google Forms, with each session taking approximately 15–20 min. Pre-test forms were sent via WhatsApp to both the experimental and control groups; however, only the experimental group received information about the study content. One week later, the experimental group participated in a 7-week group-based online training and counseling program based on the HBM, with each week focusing on a specific belief dimension. Educational content, supported by PowerPoint presentations, was shared collectively through WhatsApp between 09:00 and 17:00 and reviewed by experts. Topics addressed over the 6 weeks included sensitivity, seriousness, health motivation, mammography benefits, mammography barriers, and self-efficacy, followed by a summary video in the final week. Engagement was monitored through the WhatsApp group, and bi-weekly reminders were sent during the 3-month follow-up. All outcomes related to health beliefs, self-efficacy, and mammography behavior were self-reported. However, mammography uptake was cross-validated using official records from the Cancer Early Diagnosis, Screening, and Education Center. These records were reviewed with permission from the participating women, and mammography status was checked based on the national health database using patients’ national ID numbers, screening dates, and attendance logs. This allowed for objective verification of participants’ mammography behavior. Post-tests were administered to both groups 3 months after the training, and mammography status was verified using official screening center records. The control group did not receive any training or reminders during the study but was provided with the educational materials after the post-test.

### Data analysis

Data were analyzed using SPSS software version 22. Normality was assessed via skewness and kurtosis values (±2.0 considered acceptable). For within-group comparisons, paired-samples t-test or Wilcoxon signed-rank test was used based on distribution. Between-group differences were analyzed using independent-samples t-test or Mann-Whitney U test. Effect size was calculated using Cohen's d, interpreted as small (<0.20), medium (0.20–0.80), and large (>0.80). Statistical significance was set at p<0.05.

## RESULTS

The mean age of women in the experimental group was 46.65±4.01 years; 31.7% were primary school graduates, 87.8% were married, 31.7% were employed, and 61% reported an average perceived monthly income. In the control group, the mean age was 47.1±5.21 years; 50% were primary school graduates, 95% were married, 12.5% were employed, and 52.5% reported an average income. No statistically significant differences were found between the groups in terms of age, education, marital or employment status, or income perception (p>0.05) (Table 1).

**Table 1:** Comparison of sociodemographic characteristics of women in the experimental and control groups.

		Experimental group		Control group		Test	p
		Mean±SD	Min–Max	Mean±SD	Min–Max		
Age		46.65±4.01	41–56	47.1±5.21	41–63	t=-0.428	0.670
		**n**	**%**	**n**	**%**		
Education level							
	Literate	1	2.4	1	2.5	χ^2^=1.903	0.234
	Primary school	13	31.7	20	50.0		
	Secondary school	8	19.5	10	25.0		
	High school	12	29.3	6	15.0		
	University and above	7	17.1	3	7.5		
Marital status							
	Single	5	12.2	2	5.0	χ^2^=1.795	0.237
	Married	36	87.8	38	95.0		
Employment status							
	Employed	13	31.7	5	12.5	χ^2^=0.016	1.000
	Not employed	28	68.3	35	87.5		
Perception of monthly income							
	Income less than expenses	14	34.1	16	40.0	χ^2^=2.732	0.703
	Income equal to expenses	25	61.0	21	52.5		
	Income more than expenses	2	4.9	3	7.5		

Mean: average; SD: standard deviation; Min: minimum; Max: maximum; t: Independent-samples t-test; χ^2^: chi-square test.

After the training, the experimental group showed significantly higher mean scores across all sub-dimensions of the HBM Scale for Breast Cancer Screening compared to pre-test scores (p<0.001), while no significant change was observed in the control group (p>0.05). Effect size analyses (Cohen's d) indicated a large impact of the intervention (Table 2).

**Table 2:** Comparison of health beliefs and self-efficacy of experimental and control groups in pre- and post-tests.

			Experimental group	Control group	Difference between groups
			(Mean±SD)	(Mean±SD)	
HBM Scale for Breast Cancer Screening	Sensitivity	Pre-test	5.34±0.77	4.78±0.77	t=3.209, **p=0.002** ** d=0.8
		Post-test	6.43±2.84	4.35±2.64	t=9.372, **p<0.001** ** d=0.87
		Within-group difference	t=-6.771, **p<0.001** * d=0.95	t=-0.390, p=0.699	
	Seriousness	Pre-test	7.23±0.80	7.28±0.77	t=-0.294, p=0.769
		Post-test	10.00±3.97	7.85±1.79	t=10.784, **p<0.001** ** d=1.04
		Within-group difference	t=-12.945, **p<0.001** * d=1.61	t=-2.918, p=0.566	
	Health motivation	Pre-test	7.18±0.61	7.77±1.03	t=-3.112, p=0.343
		Post-test	10.45±2.73	7.67±0.75	t=16.752, **p<0.001** ** d=1.43
		Within-group difference	t=-21.328, **p<0.001** * d=1.65	t=0.521 p=0.605	
	Benefits of mammography	Pre-test	8.75±3.71	7.33±0.89	t=7.890, p=0.132
		Post-test	11.04±5.66	7.57±0.66	t=23.235, **p<0.001** ** d=0.87
		Within-group difference	t=-13.503, **p<0.001** * d=0.88	t=-1.429, p=0.161	
	Mammography barriers	Pre-test	6.09±0.72	7.50±1.66	t=-3.802, p=0.125
		Post-test	8.93±1.73	6.97±0.58	t=7.092, **p<0.001** ** d=0.89
		Within-group difference	t=-16.757, **p<0.001** * d=0.81	t=-8.010, p=0.546	
Mammography Self-Efficacy Scale		Pre-test	7.84±0.51	7.54±0.74	t=2.122, **p=0.037** ** d=0.47
		Post-test	10.65±4.59	7.89±0.68	t=19.391, **p<0.001** ** d=0.86
		Within-group difference	t=-19.860, **p<0.001** * d=0.87	t=-2.330, p=0.065	

Mean: average; SD: standard deviation; HBM: Health Belief Model; d: Cohen's d value.

* Paired-samples t-test;

** Independent-samples t-test. t value: paired-samples t-test (within-group comparisons); independent-samples t-test (between-group comparisons). Statistically significant values are denoted in bold (p<0.05).

Comparison of the total mean scores of the Mammography Self-Efficacy Scale revealed significantly higher scores in the experimental group both before and after the training (p<0.001). Post-training increases in the experimental group were also statistically significant (p<0.001), with large effect sizes indicating a substantial impact of the intervention (Table 2).

Following the intervention, 70% of women in the experimental group (n=29) and 32.5% in the control group (n=13) underwent mammography. Post-test results showed significant differences between the groups in the Mammography Self-Efficacy Scale and all sub-dimensions of the HBM Scale for Breast Cancer Screening, based on mammography status (p<0.001). In the experimental group, women who underwent mammography had higher scores in all sub-dimensions (except perceived barriers, which were lower) compared to the control group. Effect size analyses indicated that the intervention had a substantial impact (Table 3).

**Table 3:** The effect of mammography status on health beliefs and self-efficacy of women in the experimental and control groups after the training.

			Experimental group	Control group	Difference between groups
			(Mean±SD)	(Mean±SD)	
HBM Scale for Breast Cancer Screening	Sensitivity	Mammography	6.37±0.83	4.75±0.70	U=90.0, **p<0.001** d=2.11
		No mammography	6.55±0.90	4.91±0.62	U=81.0, **p<0.001** d=2.12
		Within-group difference	U=154.0, p=0.554	U=161.0, p=0.665	
	Seriousness	Mammography	10.01±1.01	7.52±0.67	U=93.5, **p<0.001** d=1.89
		No mammography	9.98±0.93	8.01±0.81	U=78.5, **p<0.001** d=2.43
		Within-group difference	U=166.5, p=0.829	U=108.5, p=0.052	
	Health motivation	Mammography	10.42±0.83	7.65±0.81	U=97.5, **p<0.001** d=1.77
		No mammography	10.52±0.47	7.68±0.73	U=81.0, **p<0.001** d=1.28
		Within-group difference	U=167.0, p=0.831	U=168.0, p=0.834	
	Benefits of mammography	Mammography	10.96±0.71	7.60±0.78	U=71.5, **p<0.001** d=1.51
		No mammography	11.10±0.54	7.56±0.61	U=65.0, **p<0.001** d=1.43
		Within-group difference	U=173.0, p=0.977	U=171.0, p=0.895	
	Mammography barriers	Mammography	8.01±0.61	9.12±0.58	U=59.0, **p<0.001** d=1.83
		No mammography	7.91±0.54	8.84±0.78	U=40.0, **p<0.001** d=1.14
		Within-group difference	U=157.5, p=0.635	U=144.0, p=0.362	
Mammography Self-Efficacy Scale		Mammography	10.62±0.62	7.69±0.86	U=101.0, **p<0.001** d=1.90
		No mammography	10.74±0.48	7.99±0.57	U=91.5, **p<0.001** d=1.21
		Within-group difference	U=168.0, p=0.859	U=153.5, p=0.524	

Mean: average; SD: standard deviation; HBM: Health Belief Model; U: Mann-Whitney U test; d: Cohen's d value. Statistically significant values are denoted in bold.

## DISCUSSION

In this study, the effects of HBM-based WhatsApp-supported training on women's health beliefs about mammography, self-efficacy levels, and mammography behavior were evaluated. HBM is a theoretical framework that is frequently used to explain the cognitive and motivational factors affecting individuals’ health behavior^15^. In this study, no statistically significant differences were found between the experimental and control groups in terms of age, education level, marital status, employment status, or perceived income (p<0.05) (Table 1). This homogeneity between groups strengthens the internal validity of the study, suggesting that the observed effects are due to the intervention rather than sociodemographic differences. This is consistent with previous studies indicating that comparable baseline characteristics increase the reliability of intervention outcomes^9,16,19^.

According to the findings of the study, a significant increase was observed in the perceptions of "sensitivity," "caring/seriousness," "health motivation," and "benefit of mammography," and a significant decrease was observed in the perception of "barriers to mammography" of the women in the experimental group after the training (p<0.001) (Table 2), indicating that hypothesis H_1_ was supported. Recent studies have shown that HBM-based interventions significantly increase participants’ perceptions of sensitivity, seriousness, health motivation, and benefits of mammography while significantly decreasing their perceived barriers to mammography^11–13^. The results of our study are consistent with these findings. The consistency of our study's findings with the literature suggests that the HBM-based WhatsApp-supported training applied in our study had similar positive effects. This supports the effectiveness of the model in influencing women's health beliefs and behaviors regarding mammography. Furthermore, obtaining similar results across different cultures and settings indicates the universal applicability and effectiveness of HBM-based educational interventions. These results are also supported by recent comprehensive studies that emphasize the importance of tailored screening strategies based on breast cancer stage, molecular subtypes, and demographic factors^23,24^.

Mammography self-efficacy is an individual's belief that he/she has the capacity to perform mammography screening, that he/she can succeed in having mammography, and that he/she will contribute to his/her health when he/she has mammography, and it is considered to make a very important contribution to women's adoption of mammography^14^. Studies in the literature have found that women's mammography self-efficacy increased significantly as a result of classical health education^15–17^ (p<0.001) (Table 2). The results of our study are consistent with the literature. In this study, it was determined that women's mammography self-efficacy level increased positively after the training. The H_2_ hypothesis was accepted. The fact that women underwent mammography after the training provided via the WhatsApp application supports this result.

In this study, women who received training via the WhatsApp application had higher rates of mammography than women who did not receive training (p<0.001) (Table 3). The H_3_ hypothesis was accepted. Several studies in the literature have found that women who received training on breast cancer and mammography had higher rates of mammography screening^19,20^. In our study, as seen in the related literature, education is an important factor in the acquisition of mammography behavior. It can be said that as the level of education increases, women become more conscious about health services, especially breast cancer screenings, and therefore their likelihood of having mammograms increases. The findings confirm that education, particularly when accessible and personalized through digital means, is a key driver of behavioral change. However, it is important to acknowledge a key limitation of this study: participants who were illiterate or lacked access to WhatsApp were excluded. This introduces a potential selection bias, as it may disproportionately exclude women with lower digital literacy and socioeconomic disadvantage. According to the Turkish Statistical Institute^25^, approximately 40% of women over the age of 40 in Turkey have limited digital literacy. This limitation may affect the generalizability of the findings and raise concerns regarding health equity. Future interventions should consider more inclusive and accessible formats to reach underserved populations and ensure equitable health education opportunities.

### Limitations

Since this study was conducted in only one center, it should be repeated in different populations and in different regions since cultural and social norms may affect the results. The data obtained in the study are limited to the scale used and the research group. In addition, the fact that the scales used in the study are based on self-report and the analysis is based on cross-sectional data constitutes the limitations of this study.

## CONCLUSION

This study found that HBM-based education and counseling via WhatsApp significantly improved women's health beliefs, self-efficacy levels, and regular mammography behavior. These results highlight the value of mobile-based education in nursing and health policy. It is recommended that affordable, accessible digital interventions be used to enhance women's health screening programs. The study offers guidance for health professionals in designing person-centered public health interventions that account for cultural and technological factors.

## Data Availability

The datasets generated and/or analyzed during the current study are available from the corresponding author upon reasonable request.
